# Safety of Creatine Supplementation in Active Adolescents and Youth: A Brief Review

**DOI:** 10.3389/fnut.2018.00115

**Published:** 2018-11-28

**Authors:** Andrew R. Jagim, Richard A. Stecker, Patrick S. Harty, Jacob L. Erickson, Chad M. Kerksick

**Affiliations:** ^1^Exercise and Performance Nutrition Laboratory, Department of Exercise Science, Lindenwood University, St. Charles, MO, United States; ^2^Mayo Clinic Health Systems, Onalaska, WI, United States

**Keywords:** creatine, safety, adverse events, supplementation, adolescents, youth, performance

## Abstract

Creatine has been extensively researched and is well-supported as one of the most effective dietary supplements available. There is overwhelming support within the literature regarding the ability of creatine to augment performance following short term (5–7 days) and long-duration supplementation periods. There is also strong support for creatine regarding its safety profile and minimal risk for adverse events or any negative influence on markers of clinical health and safety. Recent research has also highlighted the ability of creatine to confer several health-related benefits in select clinical populations in addition to offering cognitive benefits. Creatine is also a popular supplement of choice for adolescent athletes; however, research in this area is extremely limited, particularly when examining the safety and efficacy of creatine supplementation in this population. Therefore, the purpose of this review was to highlight the limited number of studies available in adolescent populations and systematically discuss the topic of safety of creatine supplementation in a younger population.

## Introduction

Ergogenic aids are broadly defined as any training technique, mechanical device, nutritional agent or practice, pharmacological method, or psychological technique that can improve exercise performance capacity or enhance training adaptations (1). As a dietary supplement, creatine is regarded as one of the more well-researched and efficacious nutritional ergogenic aids available to athletes (2). When supplemented in the human diet, creatine increases intramuscular creatine stores and can improve exercise capacity and training adaptations. More recently, creatine has been established as a legitimate nutritional adjunct in areas related to rehabilitation and neuromuscular disorders with growing evidence for some therapeutic efficacy in thermoregulation, concussions, head trauma, autism, and neuroprotection (2).

Creatine is a naturally occurring guanidino compound composed of two amino acids (arginine-glycine) found primarily in the flesh of animals, with the majority (~95%) being present in skeletal muscle (2). Approximately two-thirds of intramuscular creatine is phosphocreatine (PCr) while the remaining proportion is free creatine (Cr). Harris et al. (3) demonstrated that a 70-kg individual averages around 120 mM/kg dry muscle mass of total creatine (PCr + Cr) with an upper limit (after supplementation) of up to 160 mM/kg dry muscle mass. Ongoing cellular metabolism of creatine results in 1–2% being degraded, leading to a daily total (exogenous + endogenous) of 1–3 g of creatine required per day to maintain normal phosphocreatine levels. Depending on food tastes and preferences, the human diet commonly provides up to half of this amount with foods such as fish and meats being the main sources with uncooked beef and salmon delivering around 1–2 g of creatine per pound. The remaining amount of the total rate of appearance is provided by endogenous synthesis by the kidney and liver (2).

### Supplementation regimens and considerations

Creatine is stored within skeletal muscle as both free creatine and as phosphocreatine serving as a key substrate for substrate-level phosphorylation and the resynthesis of ATP (4). Collectively, these high-energy phosphates play a vital role in several metabolic processes within the body, particularly during the first 10 s of high-intensity exercise such that depletion of PCr is accepted as a major source of fatigue (5, 6). Creatine can be found in animal food sources and typically daily ingestion equates to 1–2 g per day; however ~1–2% of intramuscular creatine is non-enzymatically metabolized into creatinine daily (2). It is well-supported within the literature that exogenous supplementation of creatine is an effective strategy to increase intramuscular phosphocreatine stores by ~20–40% (2) depending on baseline levels. Commonly, a loading dose of 0.3 g/kg of body weight per day for 5–7 days is used as an effective “loading protocol,” with a subsequent daily dose of 0.03 g/kg of body weight (3–5 g/day) required for the maintenance of elevated PCr levels (3, 7, 8). Alternatively, Hultman et al. (7) demonstrated that a supplementation regimen of 3 g/day for 28 days can also result in similar levels of intramuscular creatine and phosphocreatine. Finally, once supplementation ceases, studies suggest that it may take as long 4–6 weeks before intramuscular phosphocreatine levels return to baseline (7, 9, 10). Likely due to the long half-life in muscle, studies have failed to highlight instances where endogenous production is reduced or dampened by previous creatine supplementation after supplementation is stopped. In this respect, it was commonly suggested that creatine users should cycle on and off, but continued evidence suggests that this is not necessary (11, 12) particularly when the multitude of benefits resulting from ongoing creatine supplementation are considered.

### Safety of creatine use

In adults, a growing number of published randomized controlled trials are available that support the safety of creatine supplementation. These studies have been conducted in both athletic and general populations and range from as short as a few days to as long as 5 years without any adverse changes in markers of clinical health (12, 13). Multiple studies have assessed and reported that creatine supplementation has no adverse impact on clinical health markers in competitive athletes (13–17), non-athletic populations (18–25), and in clinical populations (26–29). Furthermore, recent evidence suggests that creatine supplementation is unrelated to the formation of carcinogenic heterocyclic amines in humans, which was a long-standing concern due to creatine's potential role as a precursor of the compounds (30). Generally, the only clinically-relevant side effect of creatine supplementation is weight gain (primarily fat-free mass), which is often a desired outcome in athletes, primarily ones with an emphasis placed on strength, power and body size, and clinical patients with any type of muscle wasting disorders (2). A summary of these studies can be found in Table 1.

**Table 1:** Safety of creatine use in adults.

**Study**	**Subjects**	**Design**	**Duration**	**Dosing protocol**	**Clinical Safety measures**	**Presence of significant adverse events**	**References**
**ATHLETIC POPULATIONS**							
Armentano et al.	35 (male: 20; female: 15) active duty US army volunteers	Double-blind, placebo-controlled.	14 Days	5 g/day on Days 1–6, 20 g/day on Days 7–14	Blood and Urine Markers, Blood Pressure	None	(31)
Cancela et al.	14 male football players	Randomized, double-blind, placebo controlled	8 Weeks	15 g/day (1 week) 3 g/day (7 weeks)	Blood and Urine Markers, Hemodynamic variables,	None	(14)
Galvan et al.	Study 1: 13 recreationally-active males Study 2: 48 recreationally-active males	Study 1: Randomized, double-blind, placebo controlled, crossover. Study 2: Randomized, double-blind, placebo controlled	**Study 1:** 1 day per condition **Study 2:** 28 Days	**Study 1:** 1.5, 3, or 5 g in an acute dose **Study 2:** 6, 12, or 20 g/day for 7 days; 1.5 , 3 , or 5 g/day for 21 days	Blood Markers, Hemodynamic variables, Side effects.	None	(32)
Greenwood et al.	72 NCAA Division 1A football players	Open-label supplement intervention	120 Days	0.3 g/kg/day for 5 day, 0.03 g/kg/day for 115 day	Injury Rates, Cramping	Injury rates and cramping were significantly lower in creatine users.	(15)
Greenwood et al.	Approximately 130	Open-label supplement intervention	Mixed duration of use	15.75 g/day for 5 days, 5 g/day for remainder of intervention	Injury Rates, Cramping	Injury rates and cramping were generally lower or proportional in creatine users.	(16)
Joy et al.	58 healthy males and females	Randomized, double-blind, placebo controlled.	28 Days	1 g/day, 2 g/day	Blood Markers	None	(19)
Kreider et al.	116 healthy NCAA Division 1A football players	Longitudinal, open-label intervention.	2 Years	15.75 g/day for 5 days, 5 g/day for up to 2 years.	Blood, Serum, and Urinary Markers	None	(12)
Lugaresi et al.	26 healthy, resistance-trained males	Randomized, double-blind, placebo controlled	12 Weeks	20 g/d for 5 days, 5 g/day for remainder of trial.	Blood Markers, Markers of Kidney Function	None	(33)
Mayhew et al.	23 healthy NCAA Division 2 football players	Retrospective Design	Mixed duration of use prior to data collection	Spontaneous use	Blood Markers	None	(17)
Poortmans and Francaux	9 (male: 8; female: 1) healthy athletes	Retrospective Design	Mixed duration of use prior to data collection (10 months to 5 year)	Spontaneous use (1–80 g/day)	Blood Markers	None	(13)
Robinson et al.	48 (23 male; 25 female)	Randomized, double-blind, placebo controlled	5 days−9 weeks.	20 g/day for 5 days, 20 g/day for 5 days followed by 3 g/day for 8 weeks	Blood Markers	None	(34)
**NON-ATHLETIC POPULATIONS**							
Groeneveld et al.	175 ALS patients (male: 120; female: 55) healthy athletes	Randomized, double-blind, placebo-controlled.	Mixed duration of use; approximately 310 days.	10 g/day	Blood Markers, Side effects, Markers of Kidney Function	None	(35)
Gualano et al.	18 healthy sedentary males	Randomized, double-blind, placebo-controlled.	3 Months	10 g/day for 3 months	Blood Markers, Markers of Kidney Function	None	(18)
Gualano et al.	25 (male: 16; female: 9) Type II Diabetic Patients	Randomized, double-blind, placebo-controlled.	12 Weeks	5 g/day	Blood Markers	None	(27)
Lobo et al.	109 Osteopenic, postmenopausal females	Randomized, double-blind, placebo-controlled.	1 Year	1 g/day	Blood Markers, Adverse events.	None	(20)
Mihic et al.	30 (male: 15; female: 15) healthy adults	Randomized, double-blind, placebo-controlled.	5 Days	20 g/day for 5 days	Blood Markers, Hemodynamic variables	None	(21)
Neves et al.	26 postmenopausal females diagnosed with knee osteoarthritis	Randomized, double-blind, placebo-controlled.	12 Weeks	20 g/day for 7 days, 5 g/day for 11 weeks.	Markers of Kidney Function	None	(22)
Poortmans et al.	5 healthy males	Placebo-controlled	5 Days	20 g/day	Blood and Urine Markers, Markers of Kidney Function	None	(25)
Ropero-Miller et al.	4 (male: 2; female: 2) healthy subjects	Uncontrolled intervention	10 Days	20 g/day for 5 days, 5 g/day for 5 days	Urine Markers	None	(23)

*NCAA, National college athletic association*.

### Efficacy of creatine as an ergogenic aid

Use of creatine in athletes can be traced back to the 1990s and since that time, hundreds of papers have been published examining the impact of creatine supplementation on physical performance. In this respect, comprehensive reviews exist on this topic and the interested reader is encouraged to read them (2, 36–39). However, it is beyond the scope of this current review to discuss this literature in detail. Briefly, several studies have indicated that supplementation periods as short as 3–5 days are sufficient to confer an ergogenic benefit with improvements in exercise capacity, anaerobic capacity, power, and sport-specific tasks consistently being observed (36, 40). When supplementation stretches to several weeks, augmented training adaptations such as greater improvements in strength, lean body mass and anaerobic performance, when used in conjunction with a structured training program, are commonly reported (2, 37, 41–43). Finally, and as summarized by Kreider et al. (2), these outcomes are consistently reported across genders as well as in adolescents (44–48), younger adults (32, 49–59) and older individuals (38, 41, 60–68). Table 2 highlights many examples of sporting events that may be enhanced by creatine supplementation. As an ergogenic aid and in summary, creatine supplementation is commonly one of the most highly recommended and advocated by researchers and professional organizations (1, 2, 79).

**Table 2:** Dietary supplement use in youth and adolescent populations.

**References**	**Population**	**Key findings**
Smith and Dahm (69)	U.S. High Schools	*Population:* 328 students (55% boys, 45% girls, 15.2 ± 1.3 years) *Usage Statistics*: Creatine use of 8.2% (26 boys, 1 girl) *Sports:* Football (29%), soccer and hockey *Perceived Efficacy:* 79% felt it improved their performance *Frequency:* 35% daily, 35% weekly, 30% rate usage *Dosing:* 55% could not recall dosage, 23% (5 g/day), 23% (5–10 g/day) *Primary Information Source*: Friends
Metzl et al. (70)	U.S.—Middle and High School	*Population:* 1,103 (55% boys, 45% girls, Grades 6–12) *Usage Statistics:* Creatine use (8.8% boys, 1.8% girls). Creatine use was stable (3.4%) from grades 6–, 12% use in 11th grade and 44% use in 12th grade. *Sports:* Strength and power sports (football, wrestling, hockey) *Reasons to Take:* Improve performance (72%), Improve appearance (61%), and improve speed (40%) *Reasons Not Taking:* Safety concerns (46%), lack of perceived benefit (20%), expense (13%)
Kayton et al. (71)	U.S.—High School	*Population:* 270 athletic high school boys (45%) and girls (55%), 13–18 years *Usage Statistics:* Sports drinks were most common (59%), vitamin/minerals (46%), creatine (21% in boys, 3% in girls), amino acids (8% in boys, 1% girls) *Reasons to Take:* Gain muscle, increase energy, prevent illness *Primary Information Source:* Coaches, doctors and parents were primary sources of nutrition and dietary supplement information, parents have largest influence on use.
O'Dea (72)	Australia—Middle & High School	*Population:* 78 students, grades 7–11, 11–18 years *Usage Statistics:* Sports drink (56%), vitamin/minerals (49%), energy drinks (42%), creatine (5%), protein supplements (4%). Creatine was taken only by boys, all others were consumed by both gender. *Reasons for Taking*: Energy production or boost *Misc*.: Athletes had little to no knowledge of adverse events
Bell et al. (73)	Canada—High School	*Population*: 333 high school boys (57%) and girls (42%), 15.4 ± 1.1 years *Usage Statistics:* Vitamin/minerals (43%), protein (14%), creatine (5.3%). Boys reported similar use of vitamin/minerals as girls, greater protein and creatine use when compared to girls *Reasons for Taking*: Students taking protein (43%) and creatine (42%) believed it would help their performance
Hoffman et al. (74)	U.S.—High School	*Population:* 3,248 students in grades 8–12 *Usage Statistics:* Vitamin/minerals (59%), energy drinks (32%), protein (15%), and creatine (7%). Boys reported greater use of energy drinks, protein and creatine with progressively higher levels of protein (40% of 12th grade boys) and creatine (22% of 12th grade boys) use occurring with age. *Primary Information Source*: Teachers (36%) and parents (16%). As grade levels increased, parents, friends, coaches, athletic trainers, and internet sites take on larger roles
Petroczi and Naughton (75)	U.K.—Young Elite Athletes	*Population*: 403 elite athletes (12–21 years, 17.7 ± 2.0 years) *Usage Statistics*: Energy drinks (87%), vitamin/minerals (47%), whey protein (44%), and creatine (28%) *Sports*: Rugby, soccer and swimming *Misc*.: Large majority (78%) did not believe nutritional supplementation was needed to achieve success in sports
Diehl et al. (76)	Germany—Young Elite Athletes	*Population*: 1,138 Olympic level competitors (14–18 years, 56% boys, 44% girls) *Usage Statistics*: Vitamin/minerals (34–69%), energy drinks (64%), protein (38%), creatine (12%)
Evans et al. (77)	U.S.—Youth	*Population*: 73.7 million U.S. children (10.8 ± 0.2 years, 57% older than age of 10) *Usage Statistics*: 1.64% (1.2 million) used some form of supplement to enhance sport performance in last 30 days. 94.5% used vitamin/minerals, 44% used fish oils, 34% used creatine, 26% used fiber. *Misc*.: Boys were 2x as likely to use something. Independent of gender, usage increased with age (47% of 9–12 graders)

*Adapted from Kerksick and Fox (78)*.

## Prevalence of creatine use in adolescents

A key question to answer relative to the premise of this paper is, “Are adolescent athletes using creatine?” In this respect, the use of dietary supplements to enhance performance or health is not limited to adult populations and is an increasingly popular strategy among young, active individuals (80), as outlined in Table 3. When surveyed, ~5–20% of middle school and high school aged individuals reported taking creatine at some point. For example, Metzl and associates (70) surveyed 1,103 US girls and boys in grades 6–12 and reported that 8.8% of boys and 1.8% of girls reported supplementing with creatine. Interestingly, creatine use was consistent at 3.4% in grades 6–10 while substantially increasing to 12 and 44% use in grades 11 and 12, respectively. Similarly, Kayton et al. (71) surveyed 270 US high school students 13–18 years of age and found that 21% of boys and 3% of girls reported using creatine. When prevalence rates among adolescent athletes are evaluated, similar trends emerge as creatine is often listed as one of the more commonly used dietary supplements among this population (76, 81). Regardless, there does still appear to be variability in prevalence rates, which is likely attributable to differences in gender, sport-type and associated body composition or training-related goals (70, 75, 76, 82, 83). Males appear to be more likely than females to report using creatine and the most commonly reported reasons for supplementation often include a desire to increase lean body mass and for increased energy production. As a result, strength and power or anaerobic-type sports such as football, wrestling, and hockey appear to have the highest rates of use. For example, McGuine et al. (81) reported that 16.7% of 4,000 surveyed high school athletes reported using creatine, with prevalence rates as high as 30.1% in football players and as low as 1.3% in female cross-country runners. In a similar study, Smith and Dahm (69) reported 8.2% of surveyed high school athletes reporting taking creatine, though reported use was as high as 21% in all football players surveyed. These prevalence rates certainly highlight the fact that creatine is a popular dietary supplement choice of adolescents, highlighting the need for more research in this area.

**Table 3:** Sports and sporting events where performance may be enhanced by creatine supplementation.

**Increased PCr**
Track sprints: 60–200 m Swim sprints: 50 m Pursuit cycling
**Increased PCr Resynthesis**
Basketball Field Hockey American Football Ice Hockey Lacrosse Volleyball
**Reduced Muscle Acidosis**
Downhill skiing Water sports (e.g., Rowing, Canoe, Kayak, Stand-Up Paddling) Swim events: 100, 200 m Track events: 400, 800 m Combat Sports (e.g., MMA, Wrestling, Boxing, etc.)
**Oxidative Metabolism**
Basketball Soccer Team Handball Tennis Volleyball Interval training in endurance athletes
**Increased Body Mass/Muscle Mass**
American Football Bodybuilding Combat Sports (e.g., MMA, Wrestling, Boxing, etc.) Powerlifting Rugby Track/Field events (Shot put; javelin; discus; hammer throw) Olympic Weightlifting

*MMA, Mixed martial arts; PCr, Phosphocreatine*.

*Reproduced from Kreider et al. (2) under the terms of the Creative Commons Attribution 4.0 International License (http://creativecommons.org/licenses/by/4.0/)*.

## Review methodology

**Figure 1:**
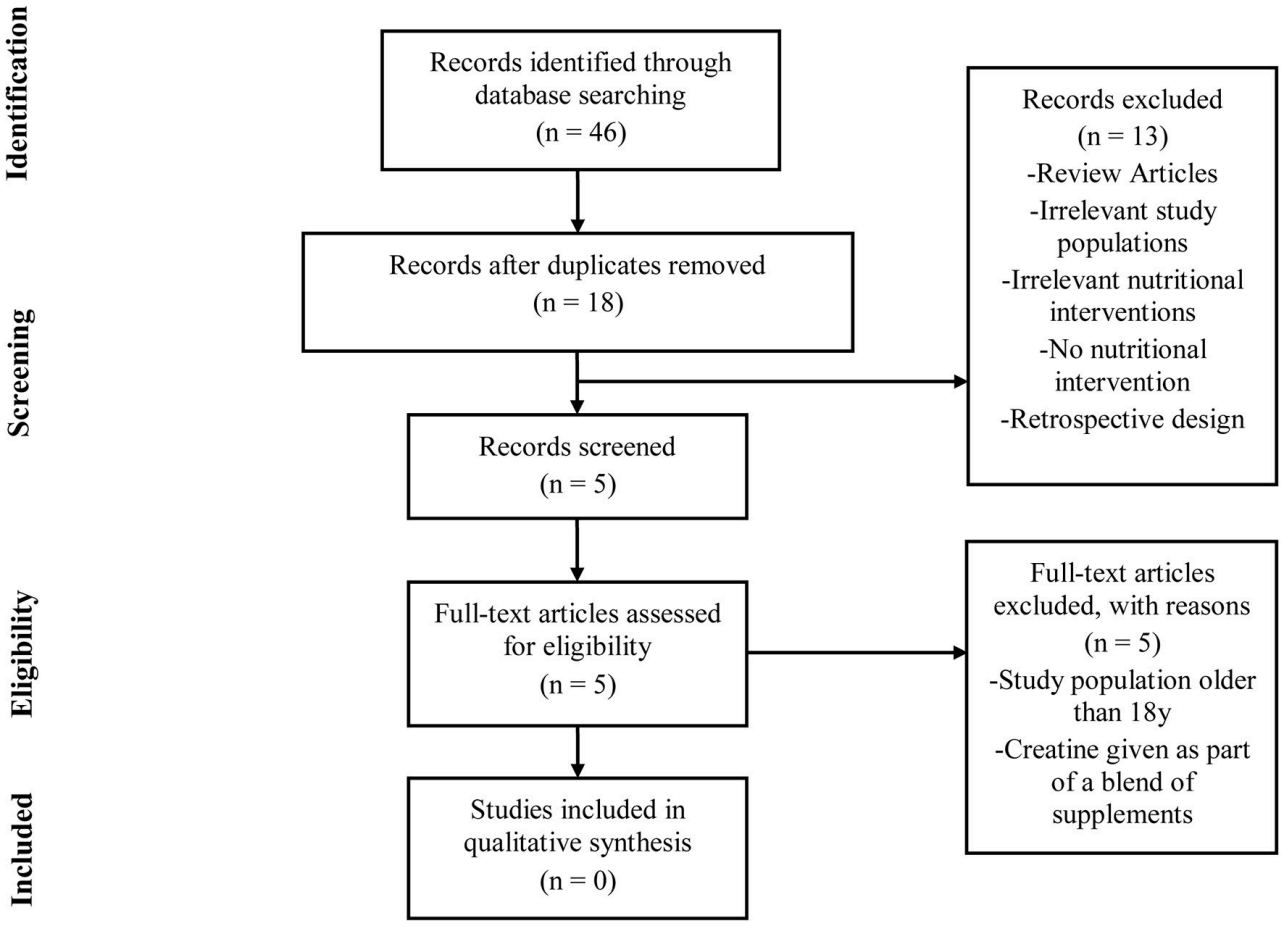
PRISMA flow chart.

To illustrate the paucity of literature directly examining the safety of creatine supplementation in youth, a systematic review was performed in accordance with the Preferred Reporting Items for Systematic Reviews and Meta-analyses (PRISMA) guidelines (Figure 1). PubMed, MEDLINE, and SportDiscus databases were each searched using the following terms: “creatine supplementation” AND “safety” AND “humans” AND “adolescents.” A second search was conducted using the terms “creatine supplementation” AND “safety” AND “humans” AND “youth.” The final search and accession date using these parameters was 08/31/2018.

Studies were eligible for inclusion if they met all of the following criteria: (1) original research conducted in humans under 18 years of age; (2) published in peer-reviewed academic journals; (3) implemented an intervention of at least 3 days using creatine only; (4) reported a clinical measure of safety as a primary outcome measure; (5) recruited populations that were not receiving creatine as a treatment for any diagnosed medical condition; (6) utilized a double-blind, placebo-controlled design. Two independent reviewers (CK and PH) assessed all articles to determine eligibility. For the purpose of this review, we have used the terms “youth” in reference to individuals between 7–12 years of age and “adolescent” for those between 13–18 years of age.

## Creatine use in youth

As illustrated, the number of published studies that have been completed in youth or adolescent populations is quite small (*n* = 8), particularly when compared to the number of studies in adults. All of the studies outlined in Table 4 used an adolescent participant and sought to examine some aspect of efficacy of creatine use in reference to sporting performance. Further, as can be seen from our PRISMA diagram (Figure 1), no published studies to date have been completed with *a priori* intent to examine the safety of creatine use in adolescents and youth. For these reasons, we have chosen to first discuss the efficacy studies available in adolescent populations before attempting to ascertain as much information as possible about the safety of creatine use in young populations.

**Table 4:** Efficacy of creatine use in adolescents.

**Study**	**Subjects**	**Design**	**Duration**	**Dosing protocol**	**Primary variables**	**Results**	**Adverse events**	**References**
Juhasz et al.	16 male Fin swimmers (15.9 ± 1.6 years)	Randomized, double-blind, placebo controlled	5 days	20 g/day	Average power, dynamic strength (swim-based tests)	↑ anaerobic performance ↑ dynamic strength.	NR	(47)
Claudino et al.	14 male Brazilian elite soccer players (18.3 ± 0.9 years)	Randomized, double-blind, placebo controlled	7 Weeks	20 g/day (1 week) 5 g/day (6 weeks)	Lower limb muscle power via CMVJ	↔ lower body power.	NR	(54)
Dawson et al.	10 males, 10 females (16.4 ± 1.8 years)	Match, placebo-controlled	4 Weeks	20 g/day (5 days); 5 g/day (22 days)	Sprint swim performance and Biokinetic Swim Bench Test	↑ swim bench test performance	NR	(45)
Grindstaff et al.	18 (male: 7; female: 11) (15.3 ± 0.6 years)	Randomized, double-blind, placebo controlled	9 Days	21 g/day	Sprint swim performance, arm ergometer	↑sprint swimming performance	None	(46)
Mohebbi et al.	17 Young soccer players (17.18 ± 01.37 years)	Randomized, double-blind, placebo controlled	7 Days	20 g/day	Repeated sprint test, dribbling performance and shooting accuracy	↑ repeat sprint performance ↑ dribbling	NR	(84)
Ostojic	20 Young, male soccer players (16.6 ± 1.9 years)	Matched, placebo-controlled	7 Days	30 g/day	Soccer specific skills tests	↑ dribble test and endurance times ↑ sprint-power test and CMVJ	None	(85)
Yanez-Silva et al.	Elite youth soccer players (17.0 ± 0.5 years)	Matched, double blind, placebo-controlled	7 Days	0.03 g/kg/day	Muscle power output (WAnT)	↑ PPO and MPO ↑ total work	None	(86)
Theodorou et al.	22 Elite (12 males: 17.7 ± 2.3 years; 10 female: 17.7 ± 2.0 years) swimmers	Randomized, double-blind, placebo controlled	11 weeks	25 g/day (4 days) 5 g/day (2 months)	Swimming interval performance	↑ Interval Performance following loading phase ↔ long-term improvements after maintenance dosing.	NR	(87)

*NR, Not Reported; ↔, Creatine supplementation resulted in no significant (p < 0.05) change; ↑, Creatine supplementation resulted in a significant increase (p < 0.05) over control*.

### Efficacy of creatine use in adolescents

Despite the overwhelming supportive body of literature regarding the efficacy of creatine supplementation in adult athletes, limited data are available in adolescent athletes. This lack of available literature is likely attributable to ethical restrictions, safety concerns and methodological challenges. With that being said, Unnithan et al. (88) eloquently described the physiological basis for creatine use in adolescents and came to the conclusion that in anaerobic athletes, there exists a metabolic rationale as to how and why creatine may provide an ergogenic benefit. Additionally, in a 2017 position stand published by the International Society of Sports Nutrition (2), it was recommended that “younger athletes should consider a creatine supplement if the following conditions are met…” and then went on to describe certain criteria surrounding approval from parents, choosing quality supplements, abiding by recommended dosing instructions and optimizing diet prior to supplementation. However, recent evidence suggests that exogenous creatine supplementation may be less effective in children and adolescents compared to adults due to limited tissue creatine uptake, particularly in brain tissues (89, 90).

Grindstaff et al. (46) were one of the first groups to examine the effects of creatine supplementation on performance in adolescent athletes. For the study, 18 male and female swimmers (15.3 ± 0.6 years) were randomly assigned to one of two groups to ingest either 21 g/day of creatine or placebo over a period of 9 days. The researchers noted significant improvements in repeat sprint performance in swimmers after creatine supplementation. Shortly after, Dawson et al. (45) tried to replicate these findings in young, elite swimmers (16.4 ± 1.8 years) using an extended creatine supplementation period of 28 days. The authors did not observe any significant improvement in single sprint performance following 4 weeks of creatine supplementation (20 g/day for 5 days; followed by 5 g/day for 22 days) but did note a significant improvement in swim bench test performance. Later in 2004, Ostojic et al. (85) observed significant improvements in soccer-specific skills following 7 days of creatine supplementation (30 g/day) in 20 young (16.6 ± 1.9 years), male soccer players. Juhasz et al. (47) examined the effects of 5 days of creatine supplementation (4 × 5 g/day) on mechanical power output and swim performance in highly trained junior (15.9 ± 1.6 years) competitive swimmers. The researchers observed significant improvements in sprint swimming performance and dynamic strength following creatine supplementation. Mohebbi et al. (84) also examined the effects of creatine supplementation (20 g/day) on repeat sprint performance, dribbling and shooting accuracy in 17 young (17.2 ± 0.1 years), soccer players. Following 7 days of supplementation, significant improvements in repeat sprint performance and dribbling abilities were observed. Most recently, in 2017 a similar study (86) with elite youth (17.0 ± 0.5 years) soccer players found significant improvements in power output following a low-dose creatine supplementation regimen (0.03 g/kg/day) for 7 days. In three of the eight studies mentioned, no adverse events or side-effects were reported by the participants following supplementation, and the remaining five studies did not report adverse event occurrences. Although not an extensive list, a precedent has been set regarding creatine supplementation interventions in adolescent athletes, warranting further research in this area examining both efficacy and safety. Table 4 outlines relevant details of these studies.

### Is creatine safe for youth?

As can be seen in Figure 1, the results of our systematic review revealed that no studies to date have been completed that sought to directly examine the safety of creatine use in an adolescent or youth population. Subsequently, each efficacy study in adolescents (Table 4) was closely reviewed by the authors to ascertain any information that might be present regarding any clinical side effects resulting from creatine use in adolescents. In this respect, none of these studies observed any gastrointestinal discomfort or changes in hemodynamic, urine, or any blood markers of clinical health and safety following the supplementation periods.

What is important to highlight is that several studies are available that have used creatine supplementation in children as some form of a medical therapy. The most common application of creatine in clinical pediatric populations involve children born with one form of a creatine deficiency syndrome. This class of syndromes results in a reduction or inability to endogenously synthesize creatine leading low levels of creatine and phosphocreatine in the muscle and brain. The physical presentation of this scenario includes muscle myopathies, gyrate atrophy, movement disorders, speech delay, autism, mental development challenges, epilepsy, and other developmental problems (91–93), as reviewed by Kreider et al. (2). Similarly, Battini et al. (94) reported on a child born with a creatine deficiency syndrome who, at 4 months of age, was treated with creatine supplementation. Moreover, Stocker-Ipsiroglu et al. (95) administered creatine monohydrate (0.3–0.8 g/kg/day, equivalent to 13.5–62 g of creatine per day for an individual weighing 45–77 kg [100–160 pounds]) to patients ranging from 25.5 months to 11 years (median age: 51 months) for a treatment period of 11–192 months (median treatment duration: 48 months). The researchers found that creatine supplementation increased brain creatine levels and stabilized other clinical outcomes. Creatine supplementation is also commonly used as a therapeutic agent for improving musculoskeletal function in patients suffering from muscular dystrophy (96–98). Most notable is a creatine supplementation study by Tarnopolsky et al. (96) who observed significant improvements in fat-free mass and handgrip strength after 4 months of creatine monohydrate supplementation (0.10 g/kg/day) in 30 young boys (mean age: 10 ± 3 years) with Duchenne muscular dystrophy. Similarly, Hayashi et al. (99) administered 0.1 g/kg creatine per day for 12 weeks to patients with childhood systemic lupus erythematosus and detected no deleterious changes in laboratory parameters of inflammation, hematology, skeletal muscle enzymes, or kidney and liver function. Creatine supplementation has also been shown to be an effective therapy to treat gyrate atrophy of the retina. Vannas-Sulonen et al. (29)reported on 13 patients who ranged in age from 6 to 31 years old. In particular, five patients were < 18 years of age (6–16 years, median: 12 years; 119–174 cm, median: 165 cm, 21–76 kg, median: 56 kg) and were treated with creatine for 40–72 months, median treatment duration: 60 months).

It is worth mentioning that some individuals point to the warnings provided on product labels that individuals younger than 18 years of age should not take creatine and inappropriately extrapolate this as evidence that creatine supplementation is unsafe in younger populations, rather than acknowledging there are insufficient data to confirm the need for such warnings. These warnings are not scientifically-based and are provided more as a legal precaution. Regardless, the point remains that no published studies are available to date that have used a rigorous study design to examine the impact of creatine supplementation on markers of health and safety in healthy populations, specifically athletes.

### A balanced perspective

Despite the overwhelming body of evidence supporting the safety and efficacy of creatine supplementation in adult athletes, there still exists a plethora of misconceptions and concerns regarding the use of creatine in adolescents that are not well-substantiated within the literature. Commonly, conversations that start with discussions of proper fueling and hydration can transition into unsubstantiated statements surrounding the purported safety of creatine use in a younger population, often associating creatine with illegal performance-enhancing drugs. For example, Greydanus and Patel (100) assessed the incidence of sports doping in adolescents and included creatine as an “anabolic-like agent” when describing anabolic steroid use. As another example, Ranby et al. (101) included creatine with anabolic steroids in the same category when describing changes in a survey designed to determine the intention or knowledge of using performance enhancing substances. These situations are troubling because first, creatine is not a steroid nor does it act like one, as it has a completely different molecular structure and physiological mechanism of action; and second, these unsubstantiated comments cloud the ability of individuals to understand key evidence-based information that exists on creatine while also instilling complications with the research process regarding adolescents. Furthermore, the toxicity from anabolic steroids are well-documented and inappropriately discussing creatine within this category implies “guilt by association.”

It is important to acknowledge that a seemingly large proportion of adolescent athletes are using or have tried creatine (see previous sections). Furthermore, it is also worth noting that creatine is not banned by any major athletic governing body or organization. While these facts are not to be intended as an endorsement of its use in young athletes, they further solidify the need for scientifically controlled investigations that seek to determine the safety of creatine use in adolescents.

## Conclusions

A major driving force of this article is to clearly focus upon the available scientific information involving creatine supplementation in youth and adolescent populations. A significant amount of concern and caution have been present within the media and sporting world up to this point regarding creatine use in younger populations. Even within the scientific literature creatine supplementation in adolescents has inappropriately been classified as “unhealthy behavior” (101), “disturbing trends” (70), or has been compared to illegal performance enhancing drug use (100) without any supporting evidence of its dangers or scientific rationale for such a classification. Unfortunately, such concern at times has resulted in multiple scenarios where dramatized accounts of creatine's impact or its associations next to other anabolic agents are made that are at best, inappropriate, and at worst, unethical. Evidence continues to accumulate; however, that clearly highlights the fact that high school-aged individuals and younger are using creatine. While prevalence statistics of creatine use in youth do not warrant an endorsement by anyone, the lack of consistent medical reports involving clinically compromising situations in combination with years of medical applications of creatine in children with inborn errors of metabolism or neurological diseases certainly opens the door for people to understand that creatine supplementation in healthy adolescent populations has the potential to be well-tolerated with little incident. Furthermore, an emerging body of literature in adolescent athletes using creatine has suggested that, first, creatine use in adolescent athletes appears to be well-tolerated with no reported adverse events and, second, that creatine use in this population can operate in an ergogenic fashion [see Table 4, also extensively reviewed by (2)]. Finally, one should not dismiss the now 25+ years of research that continues to highlight that creatine use in a multitude of populations is safe and effective means to improve both clinical and ergogenic outcomes (1).

It is our sincere hope that this article will serve as a guide for other researchers, Institutional Review Boards (IRBs), clinicians, professional organizations, and governing bodies to use when determining the safety and efficacy of creatine use in youth and adolescent populations. In this respect and in completing this review, we have identified areas where scientifically controlled, high-quality studies are needed to help build and progress this body of literature. Most importantly, short-term (<7 days) and long-term (weeks to months) studies that employ well-powered, randomized, double-blind, placebo-controlled study designs are desperately needed to examine the impact of creatine supplementation on traditional markers of clinical safety (hemodynamic changes, urine parameters, complete blood counts, and comprehensive metabolic and lipid panels) after acute and prolonged creatine supplementation regimens in adolescent populations. Therefore, this review may lend itself as a call to action for future work in this area by providing a comprehensive summary of the relevant literature and identifying the need to assess the clinical safety of creatine supplementation within this population. Such work is of paramount importance, as it will begin to demonstrate the safety of creatine use in adolescent populations under scientifically controlled conditions. From there, studies that examine the minimum effective dose of creatine or any prudent modifications to the regimens of creatine supplementation that are discussed in this article and elsewhere should be undertaken. In this respect, creatine turnover in the adult is known (and was briefly discussed), but when one considers the eating patterns of children and how creatine turnover may differ in this population, the need to explore relevant dosing amounts and patterns is also important.

## Author contributions

AJ and CK conceptualized the topic of this review. AJ, RS, PH, JE, and CK researched and analyzed the literature and assisted in manuscript preparation. JE provided scholarly guidance on the topic, assisted in manuscript preparation, and revised the manuscript critically. PH and CK performed the systematic review. All authors contributed to manuscript revision, have read, and approve of the final version of the manuscript.

### Conflict of interest statement

The authors declare that the research was conducted in the absence of any commercial or financial relationships that could be construed as a potential conflict of interest.
